# Diversity of Gut Bacteria of Field-Collected *Aedes aegypti* Larvae and Females, Resistant to Temephos and Deltamethrin

**DOI:** 10.3390/insects16020181

**Published:** 2025-02-08

**Authors:** Jennifer D. Viafara-Campo, Rafael José Vivero-Gómez, Daniel Fernando-Largo, Lina Marcela Manjarrés, Claudia Ximena Moreno-Herrera, Gloria Cadavid-Restrepo

**Affiliations:** 1 Grupo de Microbiodiversidad y Bioprospección-Microbiop, Departamento de Biociencias, Facultad de Ciencias, Universidad Nacional de Colombia, Sede Medellín, Medellín 050034, Colombia; jviafara@unal.edu.co (J.D.V.-C.); dlargo@unal.edu.co (D.F.-L.); cxmoreno@unal.edu.co (C.X.M.-H.); 2 Secretaría de Salud Departamental, Laboratorio de Entomología Departamental, Gobernación del Caquetá, Florencia 180001, Colombia; marcemanjarres@hotmail.com

**Keywords:** mosquitoes, cultivable gut bacteria, arboviruses, insecticide resistance, *Serratia*, *Cedecea*

## Abstract

The *Aedes aegypti* mosquito serves as a vector for several diseases, including dengue, Zika, chikungunya, and yellow fever. This species is well adapted to urban environments and poses a significant threat to public health. Some studies suggest that the gut bacteria of insect vectors may play a crucial role in developing resistance to insecticides. This study assessed the resistance of *Ae. aegypti* from Florencia, Caquetá, to temephos and deltamethrin and analyzed the diversity of gut bacteria in resistant larvae and adult females. Larvae exhibited resistance to temephos at a lethal concentration 50 (LC_50_) of 0.034 µg/mL, while females showed resistance to deltamethrin at a discriminant concentration of 10 µg/mL. The bacterial load in the guts of deltamethrin-treated females (3.42 × 10^6^ CFU/mL) was significantly higher compared to temephos-treated larvae (9.4 × 10^5^ CFU/mL) and untreated females (8 × 10^4^ CFU/mL). A total of sixty-eight bacterial strains were isolated from the guts of both larval and resistant females *Ae. aegypti*, with 31 strains identified through 16S rRNA gene analysis and 11 confirmed by *gyrB* gene sequencing. In untreated females, *Bacillus* comprised 12.55% of the gut bacteria and was identified as an exclusive genus. In resistant larvae, *Serratia* was the most abundant and exclusive genus, accounting for 35.29%, while in resistant females, *Cedecea* was the predominant genus, representing 66.67%. These findings suggest that gut bacteria may influence the resistance of *Ae. aegypti* to temephos and deltamethrin. Furthermore, this research provides valuable information that can be considered for the design of local vector control strategies. The results highlight new research focused on the study of insecticide tolerance and degradation within the gut microbiota of insect vectors of arboviruses.

## Simple Summary

*Aedes aegypti* is a key vector of arboviruses and poses a significant public health challenge due to its growing resistance to insecticides, complicating efforts to control disease transmission. This is a result of the increasing resistance of the species to insecticides. This study evaluated the resistance of *Ae. aegypti* larvae to temephos insecticide and adult females to deltamethrin in populations from Caquetá, Colombia. Additionally, it characterized the intestinal microbiota of these mosquitoes. The results revealed that the culturable bacteria present in deltamethrin-resistant females differed from those found in untreated females and in temephos-resistant larvae. These findings suggest that gut microbiota may play a crucial role in insecticide resistance, providing valuable information for understanding new resistance mechanisms. Furthermore, it could facilitate the development of potential strategies for bioremediation of contaminated water and soil, as well as biological vector control.

## 1. Introduction

The mosquito *Aedes aegypti* (L.) is an anthropophilic species, meaning it prefers to feed on human blood, and is known for being competitive with several arboviruses. This species is widely distributed in tropical, subtropical, and temperate regions of the world, where temperature and humidity favor its spread [1,2]. *Ae. aegypti* is also well adapted to urban habitats [3] and uses various natural and artificial breeding sites for oviposition [4].

Its versatility in inhabiting a wide variety of regions has made it one of the most representative vectors in the transmission of arthropod-borne viral (ABV) diseases. Among these, it is responsible for the transmission of viruses such as dengue (DENV) and its four serotypes DENV-1, DENV-2, DENV-3, and DENV-4 [5], as well as the Zika virus (ZIKV), chikungunya virus (CHIKV), and yellow fever virus (YFV) [6].

Although significant progress has been made in recent decades in the development of therapies and vaccines for mosquito-borne pathogens [7], the control of diseases such as dengue is primarily achieved through vector control. This approach encompasses the use of insecticides, removal of potential breeding sites, and biological control measures. These include introducing larvivorous fish, employing bacteria like *Bacillus thuringiensis* as a bioinsecticide [8], and endosymbionts such as *Wolbachia*, which interfere with reproduction through cytoplasmic incompatibility, ultimately reducing population densities [9]. These are the main strategies to control numerous diseases transmitted by insect vectors, especially populations of the genus *Aedes* [10].

Dengue virus (DENV) poses a major global public health threat, particularly in the Americas, accounting for approximately 80% of reported cases [11]. Between January and March 2024, the incidence of dengue increased by an alarming 260% compared to the previous year. Brazil, Paraguay, Argentina, Peru, and Colombia were the most affected countries [12]. This increase in cases is attributed to several factors, including population growth, migration, housing shortages, limited access to clean water, and climate change, which facilitate the global spread of *Ae. aegypti* and increase the risk of ABV transmission [13].

Specifically, the Colombian Amazon region stood out for having some of the highest incidence and mortality rates associated with dengue, with an active circulation of the four serotypes of the virus [14]. In this region, the department of Caquetá, during January 2023, the municipalities of Florencia, San Vicente del Caguán, and Solano reported the highest number of dengue cases, being the most representative municipalities for this epidemiological event [15]. Dengue represents a critical public health problem in Colombia, largely due to its high morbidity, mortality, and widespread geographic distribution. As of August 2023, a total of 67,944 dengue cases were reported, including 37,358 (55.0%) without warning signs, 29,597 (43.6%) with warning signs, and 989 (1.5%) classified as severe dengue [14].

The increase in dengue cases is also linked to the resistance of both adult and immature mosquito stages to insecticides, which represents a significant and ongoing threat to vector control programs [16]. Insecticide resistance (IR) of *Aedes* has been reported worldwide for the four main classes of insecticides, namely organochlorines (OC), carbamates (CA), pyrethroids (PY), and organophosphates (OP), but mainly against the latter two [17]. This ability is associated with well-established resistance mechanisms in different natural populations [18,19]. Previous research has demonstrated that the widespread use of insecticides for mosquito control has contributed to resistance through four different biological mechanisms: behavioral resistance, decreased cuticular penetration, modification of the chemical site of action, and metabolic resistance [10,20].

In Colombia, the increasing resistance of *Ae. aegypti* to the organophosphate larvicide temephos and the pyrethroid adulticide deltamethrin have hindered the control of this vector in some regions of the country [21]. Resistance of *Ae. aegypti* to the organophosphate temephos has been documented in several departments of Colombia, including Caquetá [22]. These populations have shown resistance to other insecticides, such as fenitrothion, lambda-cyhalothrin, and deltamethrin [22]. Given these challenges, we must advance research and develop more effective control strategies to address insecticide resistance. Recent studies have demonstrated that gut bacteria isolated from *Ae. aegypti* larvae reduce insecticide toxicity [23]. Additionally, antibiotics suppress the midgut microbiota in field populations of *Ae. aegypti*, affecting their response to pyrethroid insecticides and decreasing survival by reducing enterobacteria, which could play a significant role in resistance [24]. For example, the bacteria *Serratia oryzae* in *Ae. albopictus* may increase resistance to the insecticide deltamethrin by upregulating detoxification genes and enzymes such as carboxylesterases [20].

Eukaryotic organisms should not be considered isolated entities; instead, they are best viewed as holobionts—a host and its closely interacting community of microorganisms. This relationship affects various physiological, metabolic, reproductive, and genetic processes, highlighting the crucial role of the microbiome [25]. Among these interactions, symbiotic gut bacteria (bacteriome) play a key role, as they enhance the mosquito’s adaptability to the environment and provide immunity against pathogenic microorganisms [26]. Furthermore, some bacteria induce the production of detoxification enzymes that contribute to the host’s resistance [27]. Mosquitoes obtain their microbial communities through both vertical inheritance and environmental exposure [28].

Several studies have shown changes in bacterial composition associated with mosquito insecticide resistance. Dada et al. [29] found significant differences in the microbiota of Anopheles albimanus resistant and susceptible to the insecticide fenitrothion, observing an increase in insecticide-degrading bacteria and a lower bacterial diversity in the resistant type. In 2020, Arévalo et al. identified specific bacteria, such as Pseudomonas viridiflava, in lambda-cyhalothrin-resistant *Ae. aegypti*, suggesting that these could contribute to resistance [30]. Likewise, sequencing of the 16S rRNA gene has revealed differences in the gut microbiota of *Ae. albopictus*, showing variations in bacterial diversity and abundance among deltamethrin-sensitive and -resistant larvae [31]. Advances in both culture-dependent and culture-independent methods have enhanced our understanding of the gut microbiota of *Ae. aegypti*, indicating that it is predominantly colonized by Gram-negative bacilli, mainly of the family Enterobacteriaceae [32]. Commonly identified genera include *Enterobacter*, *Enterococcus*, *Pantoea*, *Klebsiella*, *Elizabethkingia*, *Serratia*, *Asaia*, *Sphingomonas*, *Pseudomonas*, *Acinetobacter*, and *Bacillus* [32,33,34,35].

In this study, the resistance of larvae and adult females of *Ae. aegypti* to the insecticides temephos and deltamethrin from Florencia, Caquetá, was evaluated. Culturable gut bacteria were also characterized, and the relationship between the composition of the microbiota and resistance to these insecticides was analyzed. The aim was to understand whether specific bacterial communities influence the effectiveness of insecticide treatments and contribute to resistance in these mosquito populations.

## 2. Materials and Methods

### 2.1. Ethical Declaration and Biological Permits

This project received ethical approval under CEMED certificate 18–23 (12 April 2023) from the Universidad Nacional de Colombia—Medellín.

Insect collection is covered by the Framework Permit for the Collection of Specimens of Wild Species of Biological Diversity for Non-Commercial Scientific Research Purposes, (Hermes code 59946), granted by Colombia’s National Environmental Licensing Authority (ANLA) to the Universidad Nacional de Colombia (resolution No. 0255 of 14 March 2014 (art. 3)). Larvae, pupae, and adults of *Ae. aegypti* were deposited in the Francisco Luis Gallego Entomological Museum (MEFLG) of the Universidad Nacional de Colombia (65163–65187). Bacterial isolates were deposited in the IBUN collection at the Universidad Nacional de Colombia (code IBUN-090-05490-05517).

### 2.2. Study Area

**Figure 1:**
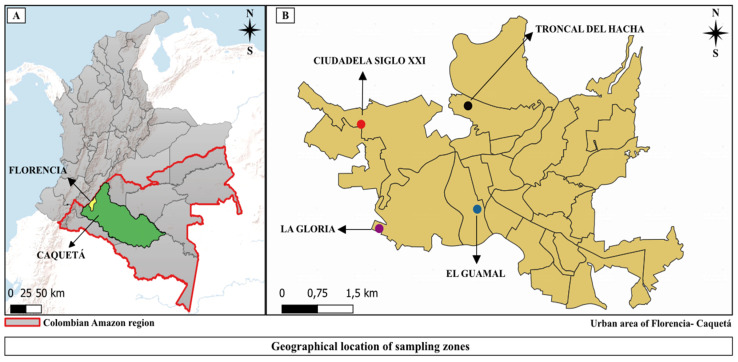
The geographical location of the study area selected for the installation of ovitraps. (**A**) Location of the city of Florencia, Caquetá, Colombia. (**B**) Location of neighborhoods in Florencia, Caquetá. QGIS, v. 3.34.12, source of layers https://datos.siatac.co/ (accessed on 12 December 2024).

The collection of *Ae. aegypti* was carried out in August 2023 (low rainfall season) in the urban area of the municipality of Florencia, Caquetá, Colombia (Figure 1A). This region is characterized by an average annual rainfall close to 3840 mm and an average altitude of 242 m.a.s.l., with temperatures ranging from 25 °C to 35 °C and a relative humidity above 80% [36]. This municipality was selected due to its high incidence of dengue cases, registering a total of 542 cases (severe dengue), which positions it as the municipality with the highest number of cases within the 1603 reported in the 16 municipalities of the Caquetá department in the Dengue Epidemiological Bulletin Week number 19 of 2023 [15].

Dwellings were selected randomly in the neighborhoods of “El Guamal” (1°36′32.7″ N, 75°36′58.2″ W), “La Gloria” (1°36′18.4″ N, 75°37′26.6″ W), “Ciudadela Siglo XXI” (1°37′25.2″ N, 75°38′10.6″ W), and “Troncal del Hacha” (1°37′45.6″ N, 75°36′57.4″ W) (Figure 1B). This selection was carried out with the collaboration of personnel linked to the Vector-Borne Diseases (TVUS) Program of the Ministry of Health of the Department of Caquetá, considering the epidemiological reports of clinical cases of dengue. In addition, a notable proliferation of mosquito vectors was reported in these neighborhoods, based on information from the general population and data from the Entomology Laboratory of the Caquetá Departmental Health Secretariat. On the other hand, the Surveillance Network for Resistance to Insecticides for Use in Public Health in Colombia, in its 2018 report, pointed out the presence of resistance in populations of *Ae. aegypti* to the insecticides temephos and deltamethrin in these localities [22].

### 2.3. Installation of Ovitraps for the Collection of Eggs of Aedes aegypti

Before the installation of the ovitraps, an awareness campaign was conducted with homeowners from the various neighborhoods, the purpose of the study was explained, and verbal consent was obtained. Black plastic ovitraps were used, each featuring a strip of rigid orange denim fabric, with dimensions of 14.5 cm long by 7 cm wide. Each ovitrap contained approximately 300 mL of water and was properly labeled with relevant information. Between 1 and 4 ovitraps per dwelling were installed randomly, for a total of 25 ovitraps per neighborhood, reaching a total of 100 ovitraps distributed in the sampled urban area, maintaining 50–100 m between them. They were placed in strategic locations, such as indoors, peridomestic areas, and green spaces, for five days, with rotations in the negatives to improve the effectiveness of the sampling. Georeferencing data (GPSmap GARMIN, 60CSx) and other variables relevant to the study were recorded.

After five days, the ovipositions were collected, washing the strips carefully to eliminate waste, external microorganisms, and even ovipositions of other insect species. The strips were then stored for transfer to the Entomology Laboratory of the Departmental Secretariat of Public Health of Florencia, Caquetá (LESSDC). There, the state and development of oviposition were verified by stereoscopic observation, using a 5% solution of hypochlorite to clarify the biological material. Once the oviposition was verified, the strips were dried at room temperature to prevent hatching and ensure viability.

Subsequently, the samples were transferred to the insectarium of the Program for the Study and Control of Tropical Diseases (PECET in Spanish) of the Universidad de Antioquia, where the egg count of each strip was carried out using a stereomicroscope (Leica, Wetzlar, Germany, model No. Z45V 120 Vac), and viable and non-viable eggs were classified into two groups. Based on information obtained from the different sampled neighborhoods, the ovitrap positivity index (OPI = No. of positive traps/No. of traps examined) × 100 and the egg density index (EDI = No. of eggs/No. of positive traps) were calculated [37]. The biological material was kept under controlled conditions (27–28 °C, a relative humidity of 50–60%, and a 12:12-h light–dark photoperiod) until the F0 and F1 generations were obtained.

### 2.4. Active Search for Immatures

An active search for larvae and pupae of *Aedes* sp., by using Pasteur’s plastic pipettes [38], was conducted in one of the selected locations (La Gloria neighborhood). The purpose of the larvae active search was to obtain adult mosquitoes to be used as a comparison with individuals treated with insecticide. The immatures were transferred to a container with water from the same artificial hatchery (artificial conduit with rainwater) and subsequently transported to the LESSDC. They were then transferred to the PECET insectarium, where they were kept until they reached their adult state under controlled conditions: 27–28 °C, 50–60% RH, and 12:12 L:D photoperiod. The morphological identification was carried out according to the taxonomic key of Rossi and Almirón [39]. The larvae were fed with commercial tetramine (TetraColor, Irving, TX, USA), while the adults were given a sterile 10% sugar solution.

### 2.5. Breeding of Immature and Adult Aedes aegypti for Resistance Bioassays

Oviposition hatching was carried out in two independent egg batches from the same brood line for bioassays with insecticides using immature stages and adult females. The first batch was intended exclusively to obtain L3–L4 larvae from an F0 *Ae. aegypti* population from Florencia. Instead of hatching the eggs separately according to the neighborhood of origin, eggs from different neighborhoods were grouped to reach the amount needed for bioassays, thus avoiding biases related to variability between egg populations from different neighborhoods. In addition, eggs of the *Ae. aegypti* Rockefeller strain, kindly donated by the PECET insectarium, were also hatched. This strain was used as a reference to test insecticide effectiveness and as an experimental control in the temephos susceptibility bioassays.

The second egg batch was the *Ae. aegypti* population from Florencia and the Rockefeller strain (control). Both groups were reared until adult state. Females of the F0 generation were maintained in entomological cages until they reached five days of age and were fed a sterile 10% sugar solution. They were then used in a CDC bottle bioassay to assess their resistance to the discriminant concentration of deltamethrin (10 μg/mL). The offspring of immature stages and adults of both populations were kept under the insectarium conditions described above. Taxonomic confirmation of larvae and adults of *Ae. aegypti* was performed using Rossi and Almirón’s key [39] and a Culicidae identification manual [40].

### 2.6. Bioassay of Susceptibility to Temephos in L3–L4 Larvae of Field-Collected Ae. aegypti

The bioassay with temephos (technical grade, 97.7% purity; LGC DR, Ehrenstorfer, Augsburg, Germany) was performed following standardized procedures by the World Health Organization (WHO) [41], with some modifications, to assess the susceptibility of field-collected *Ae. aegypti* L3–L4 larvae. Larvae were exposed to increasing concentrations of the insecticide, 0.01, 0.02, 0.03, 0.04, and 0.05 μg/mL [42], distributed in 6-well plates, each with a total volume of 10 mL. Four wells were intended for replicates, and the remaining two wells were used for positive and negative controls (Figure S1A). The plates were kept covered with their respective lids throughout the experiment to minimize evaporation and maintain a constant water volume. For the concentrations 0.01, 0.02, and 0.03 μg/mL, F0 larvae were used, while F1 larvae were used for the higher concentrations. The discriminant concentration of temephos for monitoring insecticide resistance, as recommended by the WHO, is 0.012 mg/L [43].

Twenty L3–L4 larvae of *Ae. aegypti* from Florencia, Caquetá, were placed in each well. Positive controls consisted of larvae from the susceptible Rockefeller strain exposed to different concentrations of insecticide. Negative controls included field-collected mosquitoes exposed to the insecticide solvent absolute ethyl ethanol (Biopetrolabs, Bogotá, Colombia). Before the trial, wells were prepared with the respective insecticide concentrations in dechlorinated water. Mortality was assessed 24 h after exposure, and the mortality percentage was calculated along with the mean lethal concentration 50 (LC_50_), using the R Studio software (4.1.0, 2024) with the ggplot for data analysis. A logistic regression model was applied to adjust mortality data based on insecticide concentration, enabling the estimation of the LC_50_, which indicates the concentration necessary to cause 50% larvae mortality. The dose–response relationship was graphically represented using R Studio’s ggplot2 package.

A resistant larvae fraction was used for gut extraction, while the rest were maintained until they reached the adult stage. Viable adult mosquitoes (F0) were reared under controlled laboratory conditions and blood-fed, resulting in two ovipositions. The L3–L4 larvae from these ovipositions were used for bioassays under 0.04 and 0.05 μg/mL temephos concentrations.

Resistance was assessed using the WHO criteria to categorize populations as follows: susceptible when mortality was ≥98%; between 90% and 97% suggests the possibility of resistance, requiring confirmation; and mortality < 90% indicates resistance [44].

### 2.7. Centers for Disease Control (CDC) Bottle Bioassay for Susceptibility Evaluation to Deltamethrin

For the CDC bioassay, deltamethrin from the insecticide deltamethrin (technical grade, 98.6% purity; Sigma-Aldrich, St. Louis, MO, USA) was used, and the discriminant concentration of 10 μg/mL was evaluated, following the protocol already established by the Pan American Health Organization [45]. In summary, the protocol uses 150 5-day-old adult female F0 mosquitoes fed sterile 10% sugar solution. Twenty-five mosquitoes were placed in each of the six bottles used in the test, after two hours of previous fasting of the mosquitoes. Four experimental replicates were established, along with two controls (positive and negative) in 250 mL Wheaton bottles (Figure S1B). For controls, 1 mL of acetone (LiChrosolv, Darmstadt, Germany) was applied in the negative control and 1 mL of deltamethrin in the positive control. The same amount of insecticide was applied to the bottles intended for the exhibition, and the bottles were allowed to dry for one day before being used in the bioassay. The females were introduced into the bottles, starting with the control ones and then followed by the insecticide-treated bottles.

The exposure period lasted 30 min, after which the number of fallen and dead mosquitoes was immediately recorded following the WHO mortality criteria. The mosquitoes were then transferred to entomological cages, separated by replicate, and fed with a 10% sterile sugar solution. After 24 h, the number of live (10) and dead (90) mosquitoes was recorded to determine whether the population was susceptible or resistant to the insecticide. The resistance status was determined according to the WHO criteria after a 30 min exposure period and a 24 h recovery period [46]. Mortality rates between 98% and 100% indicate susceptibility, rates between 90% and 97% suggest potential resistance requiring confirmation, and rates below 90% suggest that the population is resistant to the tested insecticide.

### 2.8. Gut Dissection of Ae. aegypti

The specimens were surface-sterilized individually with saline and 70% ethanol. The guts of mosquitoes and larvae were dissected under sterile conditions using 1X PBS, under a dissecting binocular microscope (Motic SMZ 168).

The guts of L3–L4 larvae and females of *Ae. aegypti* (Figure S1) were obtained following the methodology described by Vivero et al. [47]. Subsequently, they were transferred to a 1.5 mL microcentrifuge tube containing 200 μL of 1X PBS and homogenized with a sterilized micropestle. Fifteen guts were obtained from untreated females, which were divided into three groups of five guts each. Seventeen guts were obtained from temephos-resistant L3 and L4 larvae (0.01 to 0.05 μg/mL) and grouped into 14 pools (five for each pool), except at the concentration of 0.05 μg/mL, where two pools were formed (five for each pool). In the case of deltamethrin-resistant females, ten guts were obtained and grouped into two pools (five guts per pool).

### 2.9. Isolation and Characterization of Bacteria from Mosquitoes

The homogenized lysate from *Ae. aegypti* larvae and females was serially diluted in 1X PBS (20 μL) to prepare a serial dilution from 10^−1^ to 10^−3^. A 100 μL volume from each dilution was plated on the surface of Luria–Bertani (LB) agar (Hardy Diagnostics, Santa Maria, CA, USA) and incubated at 37 °C for 24 to 48 h under aerobic conditions (Figure S2). Environmental and 1X PBS controls were included to rule out contamination.

Microbial growth was assessed based on the total number of colony-forming units (CFUs). Bacterial colonies were subcultured to obtain pure cultures, and microbiological characterization was performed, including macroscopic (i.e., color, size, shape, and surface) and microscopic (Gram staining) characteristics. Strains were cryopreserved in 25% *v*/*v* glycerol at −80 °C for subsequent molecular analysis [47].

To evaluate differences in CFU/mL counts among untreated females (UFTs), temephos-resistant larvae (TRL), and deltamethrin-resistant females (DRFs), an analysis of variance (ANOVA) was performed using RStudio software. The *aov* function was used to compare group means, with a significance level of *p* < 0.05 to determine statistical significance. If the ANOVA showed significant differences, a post hoc Tukey test (Tukey HSD function) was conducted to identify specific differences between treatments. Additionally, a box plot was generated using the ggplot2 library to visualize the distribution of CFU/mL counts for each treatment.

#### 2.9.1. Molecular Characterization

Bacterial DNA was extracted from each pure culture using the PowerSoil^®^ DNA isolation kit (MO BIO Labs, Carlsbad, CA, USA) following the manufacturer’s guidelines. The quantification and evaluation of DNA quality was performed using a NanoPhotometer N60 (IMPLEN, Munich, Germany).

Template DNA from all isolates was initially evaluated by amplifying the inter-ribosomal region (ITS) using primers L1 (5′-CAAGGCATCCACCGT-3′) and G1 (5′-GAAGTCGTAACAAGG-3′) [48], as previously described by Moreno et al. [49].

The DNA isolated from selected colonies was used to amplify the 16S rRNA gene (1.5 kbp), using the primers 27F (AGAGTTTGATCCTGGCTCAG) and 1492R (GGTTACCTTGTTACGACTT) [49]. Both reactions (20 μL) contained 1X buffer, MgCl_2_ (2 mM), dNTPs (0.2 mM), primers (0.25 μM), Taq polymerase (0.05 U/μL), ultrapure water, and approximately 14 ng of DNA.

The thermal profile for both amplifications included an initial denaturation step at 94 °C (1 min), followed by 35 cycles at 94 °C (15 s), 58 °C (1 min), 72 °C (1:30 min), and a final extension at 72 °C (7 min). For the ITS region, an additional initial step at 72 °C (1:05 min) was included. The amplification products were visualized by electrophoresis on agarose gels (2% for ITS and 1.2% for 16S rRNA) stained with EZ Vision™ fluorescent marker and photographed under UV light using the System UV-Transilluminator V10-Uvitec. Bacterial isolates were selected based on macroscopic, microscopic, and molecular characteristics, including ITS banding profiles and sample origin.

To confirm the taxonomic identity of some bacterial isolates, the *gyrB* gene (1200 bp), which encodes the β subunit of DNA gyrase [50], was amplified. Primers UP1F (5′–AGC AGG GTA CGG ATG TGC GAG CCR TCN ACR TCN GCRTCN GTC AT−3′) and UP2R (5′–GAA GTC ATC ATG ACC GTT CTG CAY GCN GGNGGN AAR TTY GA−3′) were used [51]. The 20 μL PCR reaction included 1X buffer, MgCl_2_ (1.5 mm)*,* dNTPs (0.2 mM), primers (0.25 µM), *Taq* polymerase (0.06 U/μL), ultrapure water, and DNA with an average concentration of 28 ng. The thermal profile used consisted of an initial cycle at 94 °C (5 min), followed by 30 cycles of 94 °C (1 min), 53 °C (1 min), and 72 °C during (1:30 min), and 1 cycle of 72 °C (7 min) [51]. The DNA of *Bacillus* sp., avocado thrips, and ultrapure water were used as positive and negative controls, respectively. The amplification product was analyzed by electrophoresis in a 1% agarose gel for 40 min at 80 volts.

#### 2.9.2. Sequencing and Phylogenetic Analysis of Gut Bacteria

The amplification products of the two gene markers 16S rRNA and *gyrB* were sequenced in both directions using the ABI PRISM 3700 DNA analyzer service of Applied Biosystems. For the construction of phylogenetic trees, reference sequences of these genes were obtained from the NCBI RefSeq database, choosing those with the highest similarity for each of the samples evaluated. The sequences were aligned using the Muscle method [52] in MEGA 11 [53]. In the final alignments, sites with gaps were excluded, considering only variable positions. For the construction of the phylogenetic tree, IQ-TREE 2.0.7 was used [54], and it was inferred using the maximum-likelihood method. This software allowed the selection of the best substitution model using ModelFinder [55]. For the sequence alignment of the 16S rRNA marker gene, the Kimura model was used, with 2 parameters and a gamma distribution of 4 categories (K2P + G4) [56]. In the case of the *gyrB* gene, the Tamura–Nei model [57] was used with empirical base frequencies and a gamma distribution of 4 categories (TN + F + G4). The consensus tree was constructed from 1000 bootstrap replicates using the method described by Hoang et al. [58]. *Campylobacter upsaliensis* (CCUG 14913, NZ OU701459.1) was used as outgroups for phylogenetic analysis of the 16S rRNA and *gyrB* genes, respectively.

16S rRNA gene partial sequences were deposited in GenBank under the following accession codes: PP731791, PP732996, PP708950, PQ083278, PQ083526, PQ083832, and PQ083846.

*gyrB* partial sequences are available with the following access codes: PQ538553–PQ538561.

#### 2.9.3. Diversity of the Bacterial Community

A PERMANOVA (multivariate permutation similarity analysis) was performed to assess differences in species composition between treatments (UFTs, DRFs, and TRL) using abundance data of the identified species. This analysis was performed in Rstudio, using the Vegan package. To compare the treatment species composition simultaneously, the vegdist function was used to calculate the Bray–Curtis dissimilarity matrix and the adonis2 function to perform the PERMANOVA analysis.

Bacterial species composition data were calculated using Past software (version 4.04). A multivariate analysis was performed using the principal component sorting (PCA) function to reduce the dimensionality of the data. The PCA results, which included the coordinates of the first three main components (PC1, PC2, and PC3) for each species, were exported in a format suitable for further analysis in R studio. To visualize the PCA, the ggplot2 package was used to create a PCA biplot representing the first two main dimensions (PC1 and PC2).

## 3. Results

### 3.1. Index of Positivity, Viability, and Egg Density (HDI) in Ovitraps in Florencia, Caquetá

The positivity of ovitraps ranged from 60% to 100%. The neighborhoods with the highest rates were Guamal (100%) and La Gloria (88%), while the lowest rates were observed in Troncal del Hacha (68%) and Ciudadela Siglo XXI (60%) (Figure S3).

Overall, 79% of ovitraps had ovipositions of *Aedes* sp., while 21% were negative during the sampling period. A total of 6239 *Aedes* sp. eggs were collected, of which 5417 (86.82%) were viable and 822 (13.18%) were non-viable (Figure S4). Of the total eggs collected, 2447 (39.22%) were from La Gloria, 2192 (35.13%) were from Guamal, 848 (13.60%) were from Troncal del Hacha, and 752 (12.05%) were from Ciudadela Siglo XXI.

**Figure 2:**
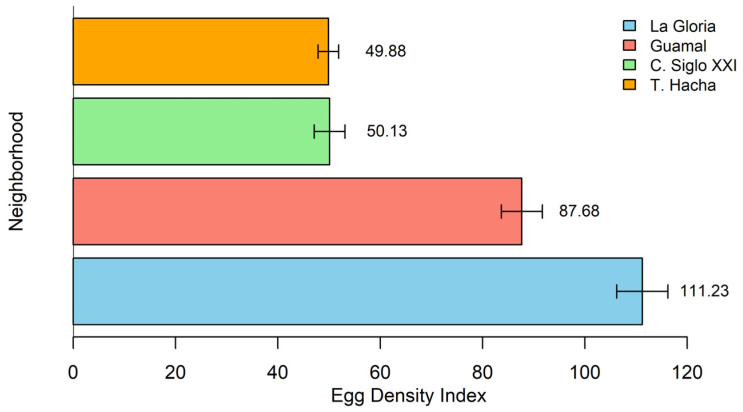
Egg density index (EDI) by neighborhood. La Gloria has the highest EDI (37.22%), and Ciudadela Siglo XXI and Troncal del Hacha have the lowest (16.77% and 16.68%).

Regarding the egg density index (EDI) by neighborhood, there was a variation between 16.68% and 37.22%. La Gloria obtained the highest percentage with 37.22% (EDI = 111.23), followed by Guamal with 29.33% (EDI = 87.68). Ciudadela Siglo XXI and Troncal del Hacha registered the lowest percentages, with 16.77% (EDI = 50.13) and 16.68% (EDI = 49.88) (Figure 2). The maximum number of eggs collected in a single ovitrap was 309. All ovipositions were selected and combined strategically.

### 3.2. Determination of Mortality and LC_*50*_ from Ae. aegypti to Temephos and Deltamethrin

**Figure 3:**
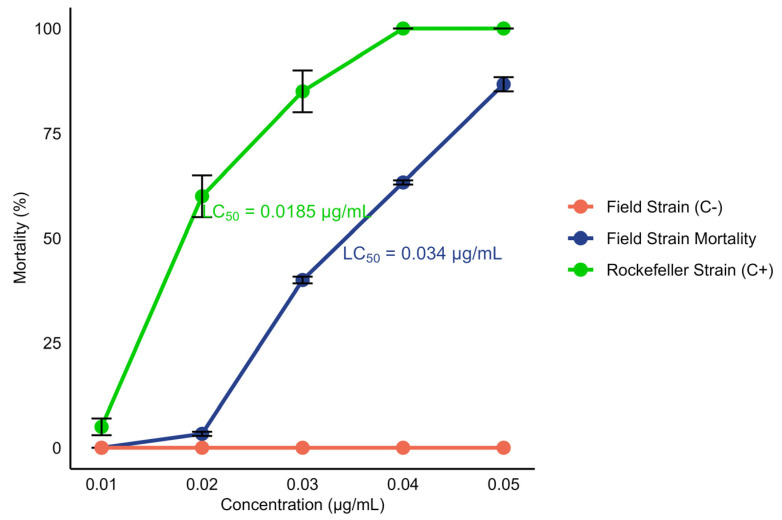
Mortality percentage of L3–L4 *Ae. aegypti larvae* after 24 h of exposure to the insecticide temephos as a function of different concentrations (0.01, 0.02, 0.03, 0.04, and 0.05 μg/mL). Positive controls (Rockefeller strain) and negative controls (field strain: not exposed to the insecticide) are included. The dots on the graph represent the average of four replicates for each concentration. The LC_50_ for the field strain was 0.034 μg/mL, and that for the Rockefeller strain was 0.0185 μg/mL.

When evaluating the susceptibility of field L3–L4 larvae of *Ae. aegypti* to temephos, a dose–response relationship was observed. The lethal concentration 50 (LC_50_) of 0.034 μg/mL was determined, which caused the death of 50% of the exposed larvae within 24 h (Figure 3).

**Figure 4:**
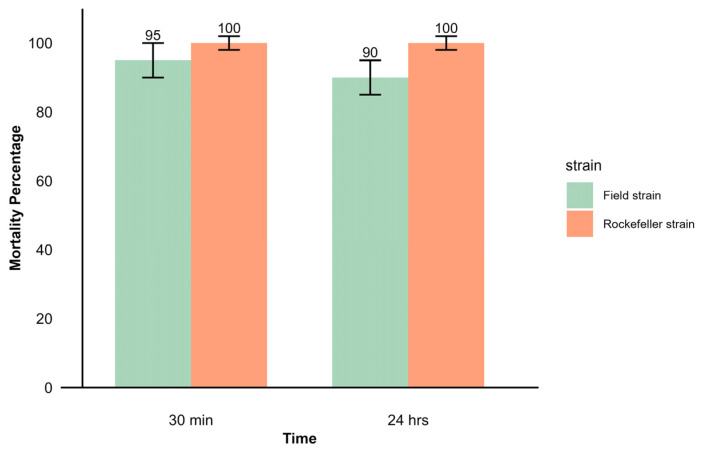
Mortality percentage observed in CDC bioassay with adult females of *Ae. aegypti* from the field and Rockefeller strains. After a 30 min exposure to the discriminant concentration of deltamethrin 10 μg/mL, mortality was recorded 24 h after recovery.

Using the CDC method, the field strain exhibited 95% mortality after 30 min of exposure to deltamethrin, which decreased to 90% after 24 h of recovery. The mortality percentages observed suggest possible resistance in the evaluated population. In comparison, the control strain recorded 100% mortality both after 30 min of exposure and after 24 h, confirming the efficacy of the insecticide on the susceptible strain (Figure 4).

### 3.3. Effects of Temephos in the Development of Ae. aegypti Larvae and Adults

In the temephos insecticide susceptibility bioassay, 400 L3–L4 larvae of *Ae. aegypti* (F0–F1) were tested. Among these, 248 larvae (62%) showed resistance at concentrations ranging from 0.01 to 0.05 μg/mL. From this group, the guts of 70 larvae (28.22%) were extracted, while 101 (40.72%) were kept under controlled laboratory conditions, excluding those exposed to 0.05 μg/mL, to evaluate their development into the adult stage. Of these 101 larvae, 93 (92.07%) reached the adult stage; however, 8 (7.9%) exhibited signs of death. In contrast, 12 larvae that were not exposed to the insecticide all developed into adults, without any mortality recorded.

### 3.4. Bacterial Diversity Through Culture Assays

#### 3.4.1. Isolation and Culture of Bacteria Isolates

Bacterial growth was monitored in 25 adults and 75 larvae that were processed in groups of five insect pools containing five specimens (Table S1). The mean bacterial count ranged from 8 × 10^4^ to 3.42 × 10^6^ colony forming units (CFU/mL). Analysis of variance (ANOVA) revealed significant differences in CFU/mL counts between treatments, with an F-value of 5.408 and a *p*-value of 0.0195, indicating that at least one of the treatments had a significantly different count. Tukey’s post hoc test identified that DRF treatment showed a significantly higher count compared to UFTs (mean difference = 1.8481, *p* = 0.0162). However, no significant differences were found between TRL and UFTs (mean difference = 0.9078, *p* = 0.1017) or between DRFs and TRL (mean difference = 0.9402, *p* = 0.1606). Treatment with deltamethrin-resistant females (DRFs) resulted in a significantly higher CFU/mL count (3.42 × 10^6^) compared to untreated females (UFTs) (8 × 10^4^) and temephos-resistant larvae (TRL) (9.4 ×10^5^) (Figure S5).

#### 3.4.2. Abundance of Morphotypes in Bacterial Cultures According to Treatment

A total of 68 bacterial isolates were obtained from LB agar cultures, distributed in the three treatments: 12 isolates from untreated females (UFTs), 49 from temephos-resistant larvae (TRL), and 7 from deltamethrin-resistant females (DRFs). Common morphotypes were observed between untreated females and resistant larvae. Microscopic characterization showed that, in the UFT treatment, 11 (92%) of the isolates were Gram-negative coccobacilli, while 1 (8%) was a Gram-positive bacillus (Figure S6A). In the DRF treatment, five (71%) of the isolates corresponded to Gram-negative coccobacilli and two (29%) to Gram-negative bacilli (Figure S6B). Finally, all isolates from the TRL treatment (100%) were identified as Gram-negative coccobacilli (Figure S6C).

#### 3.4.3. Identification of Bacterial Isolates Using 16S rDNA and *gyrB* Sequencing

A total of 35 bacterial isolates were selected using the microbial and molecular characterization of the ITS region: 8 from UFTs, 22 from TRL, and 5 from DRFs. 16S rRNA gene PCR was performed for these isolates, successfully amplifying 31 samples, 8 from UFTs, 20 from TRL, and 3 from DRFs. Three sequences from the TRL treatment were excluded from the analysis due to low quality.

**Figure 5:**
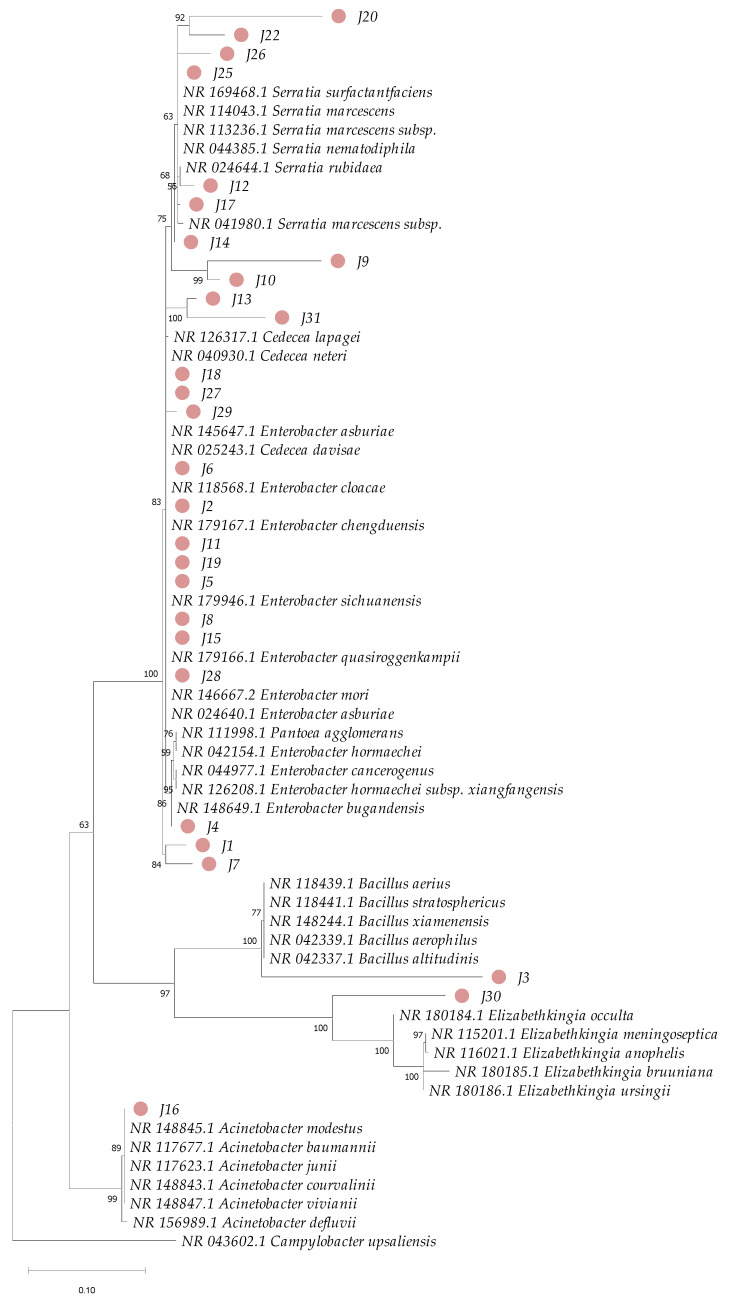
Phylogenetic analysis based on partial sequences of the 16S rRNA gene of bacteria isolated from the gut of *Ae. aegypti* and reference sequences from GenBank. Accession numbers are provided for each sample. The dendrogram was constructed using the maximum-likelihood method and 1000 bootstrap replicates. The numbers next to the branches represent the bootstrap value supporting each clade, and the pink circles highlight the isolates from this study.

**Figure 6:**
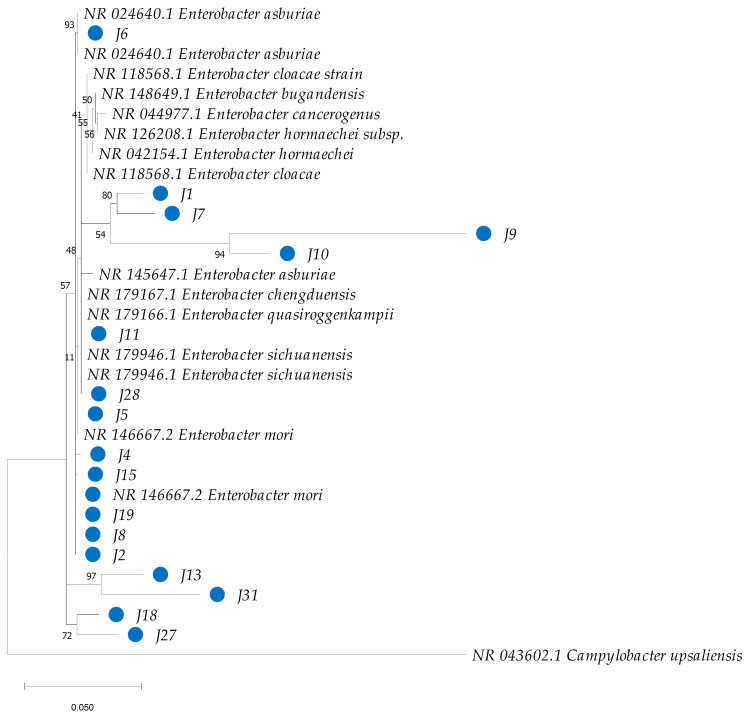
Phylogenetic analysis of partial sequences of the 16S rRNA of bacteria isolated from the gut of *Ae. aegypti*, together with reference sequences of species of the genus *Enterobacter* from GenBank. The dendrogram was constructed in MEGA with the neighbor-joining (NJ) method and a maximum-likelihood analysis, and the robustness of the clades was evaluated with a bootstrap of 1000 replicates. GenBank accession numbers are detailed in the corresponding clades. The blue circles highlight the isolates from this study.

**Figure 7:**
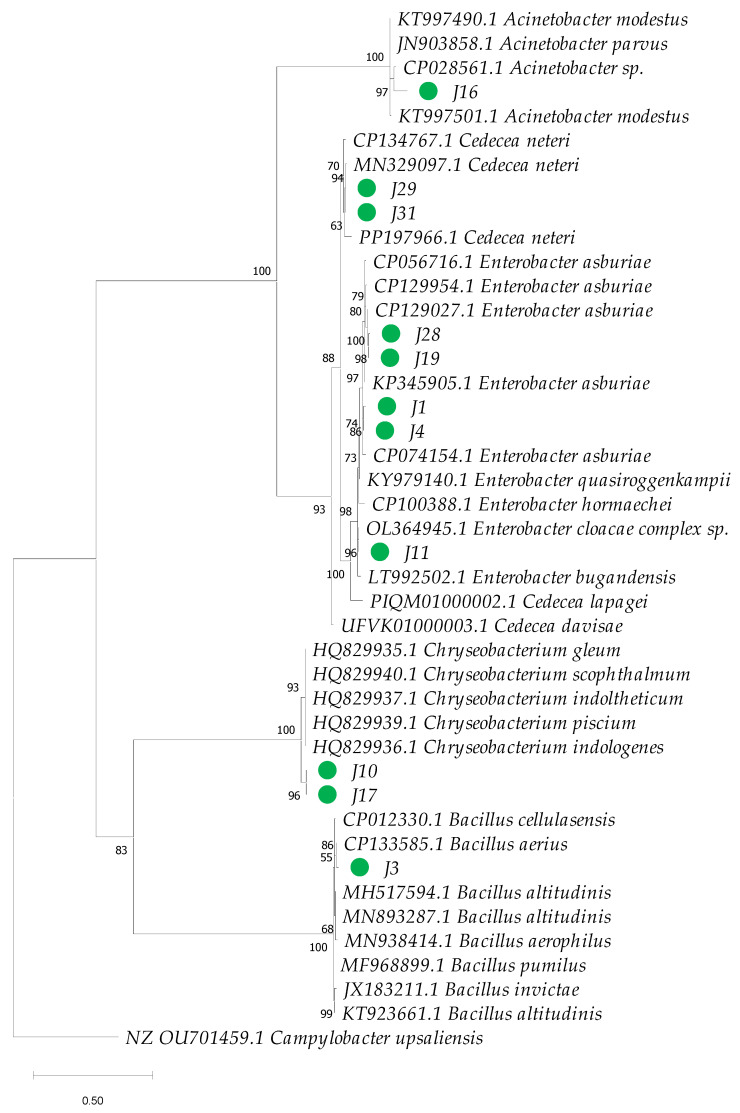
Phylogenetic analysis based on partial sequences of the beta subunit of DNA gyrase gene (*gyrB*) of bacteria isolated from the gut of *Ae. aegypti* and GenBank reference sequences. GenBank accession numbers are provided for each sequence. The dendrogram was constructed using the maximum-likelihood method in IQ-TREE, applying the Tamura–Nei model and a bootstrap analysis with 1000 replicates. The numbers next to each branch represent the bootstrap value for each clade, and the green circles highlight the isolates from this study.

Phylogenetic analysis based on 16S rRNA sequences revealed clustering patterns, with most clusters supported by bootstrap values ranging from 75% to 100% for the bacterial isolates under investigation (Figure 5). However, species-level identification within the genus *Enterobacter* proved particularly challenging due to the high genetic similarity among species within this genus (Figure 6). To overcome this limitation, additional phylogenetic analysis using the *gyrB* gene marker was conducted, enabling more precise species-level identification for 11 strains of *Enterobacter*, *Bacillus*, *Cedecea*, and *Acinetobacter* (Figure 7 and Table 1). Bootstrap values ranging from 94 to 100% further sustained the taxonomic resolution of isolates such as *Enterobacter asburiae*, *Enterobacter cloacae*, *Cedecea neteri*, *Acinetobacter* sp., *Chryseobacterium gleum*, and *Bacillus aerius*.

**Figure 8:**
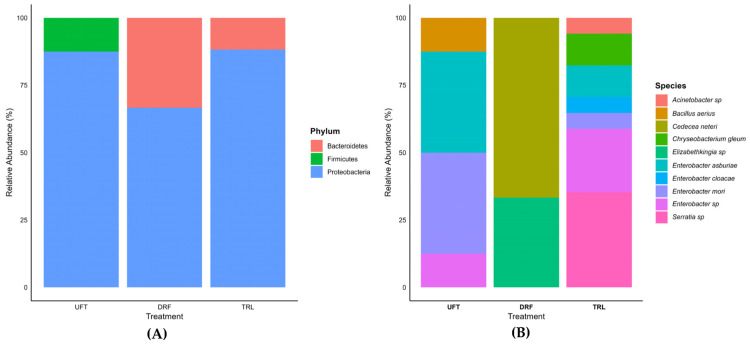
Histogram of relative abundance of bacterial phylum (**A**) and genus and species (**B**) molecularly identified in the gut of *Ae. aegypti*. The treatments include insecticide-untreated females (UFTs), deltamethrin-resistant females (DRFs), and temephos-resistant larvae (TRL).

Three main bacterial phyla were identified: *Proteobacteria*, *Firmicutes*, and *Bacteroidetes* (Figure 8A). Among these, *Proteobacteria* was the dominant phylum across all treatments, comprising 87.5% in UFTs, 66.67% in DRFs, and 88.24% in TRL, with a higher representation in resistant larvae. *Firmicutes* were found exclusively in UFTs (12.5%), while *Bacteroidetes* were detected only in DRFs (33.33%) and TRL (11.76%).

At the genus level, seven bacterial genera were identified: *Acinetobacter*, *Elizabethkingia*, *Cedecea*, *Bacillus*, *Chryseobacterium*, *Serratia*, and *Enterobacter*. The genus *Enterobacter* was the most abundant, representing 87.5% of the bacteria in the UFT treatment and 47.1% in the TRL treatment, but was not detected in DRFs. In UFTs, *Bacillus* was exclusive to this treatment, with 12.5%. In TRL, exclusive genera such as *Chryseobacterium* (11.7%), *Serratia* (35.3%), and *Acinetobacter* (5.8%) were identified. In contrast, in DRFs, *Cedecea* (66.7%) and *Elizabethkingia* (33.3%) were identified. Six different bacterial species were identified: *Enterobacter asburiae*, *Enterobacter mori*, *Bacillus aerius*, *Cedecea neteri*, *Chryseobacterium gleum*, and *Enterobacter cloacae* (Figure 8B). In DRFs, *Cedecea neteri* (66.7%) and *Elizabethkingia* sp. (33.3%) were identified, both of which were exclusive to this treatment. These results reveal significant variability in bacterial composition between treatments, with *Serratia* sp. being the most abundant genus in TRL and *Cedecea neteri* a species dominating in DRFs.

### 3.5. Bacterial Composition in Ae. aegypti Treated and Not Treated with Insecticide

The PERMANOVA analysis revealed significant differences in the composition of bacterial species among treatments. The UFT treatment accounted for 19.8% of the total variation (R^2^ = 0.19852), with an F-value of 4.0187 and a significance level of *p* = 0.022. This indicates that the microbiota composition in the UFT group significantly differed from the other treatments.

The DRF treatment had a more pronounced effect on bacterial composition, explaining 35.5% of the total variation (R^2^ = 0.35489), with an F-value of 7.1840 and a highly significant *p* = 0.002. This suggests that the microbiota composition under DRF treatment displayed the most substantial differentiation.

In the case of temephos-resistant larvae (TRL), the treatment explained 15.02% of the total variation (R^2^ = 0.15018), with an F-value of 3.0402 and *p* = 0.034. Although statistically significant, the effect was weaker than that observed for UFT and DRF treatments.

**Figure 9:**
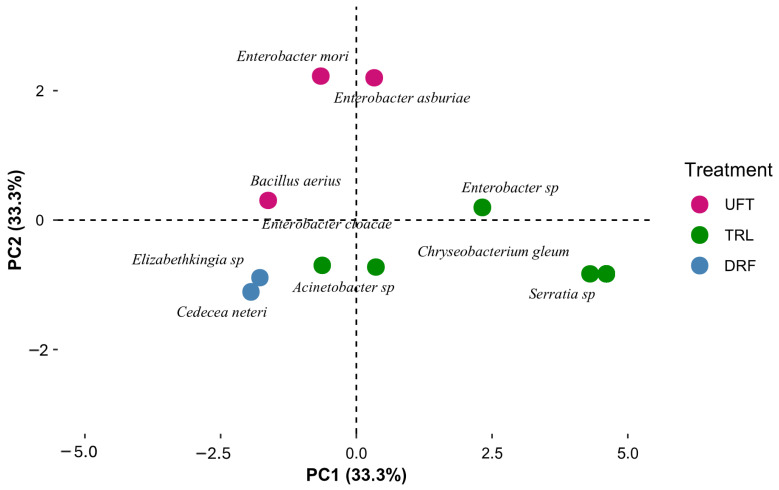
Principal component analysis (PCA) showing the distribution of gut bacteria species between untreated females (UFTs), resistant females (DRFs), and resistant larvae (TRL) of *Ae. aegypti*.

The principal component analysis (PCA) further supported these findings, showing a clear separation in bacterial composition between insecticide-treated and untreated groups of *Ae. aegypti.* The first two principal components explained 66.6% of the total variability, highlighting a distinct differentiation in the bacterial community structure associated with each treatment group (Figure 9).

## 4. Discussion

Insecticide resistance in *Ae. aegypti* represents a growing problem worldwide that compromises the efficacy of ABV control strategies and has been associated with genetic changes that affect their susceptibility [59]. However, following evidence of microbiota-mediated insecticide resistance in agricultural pests, recent studies have suggested that the gut microbiota of mosquitoes also plays a crucial role in insecticide resistance [29]. This is associated with the presence of specific bacterial species in the gut of these mosquitoes, such as *Serratia oryzae*, which is capable of degrading deltamethrin [20,60]. This provides new insights into resistance mechanisms and the role of microbiota in resistant mosquito populations [29].

The collection of ovipositions from *Ae. aegypti* using ovitraps in Florencia, Caquetá, showed a high oviposition activity, with variation among sampling locations. The high positivity observed (60%–100%) in ovitraps located inside the home suggests highly favorable conditions in the studied areas for mosquito proliferation. This result is related to the affinity of *Ae. aegypti* for the human environment, where the presence of stagnant water and certain deficiencies in infrastructure, together with water storage practices, contribute to creating a favorable reproduction environment. The presence of this vector species indicates a potential risk of transmission of ABV, such as dengue, in these domestic areas [61,62,63].

In addition, the EDI reflects that a neighborhood may have fewer positive ovitraps but a higher density of eggs, as observed in the La Gloria neighborhood and Ciudadela Siglo XXI. This confirms that a high egg density index not only reflects environmental conditions but can also be influenced by insecticide resistance. The repeated use of insecticide, without elimination of breeding sites, can select for resistant individuals [64], allowing mosquito populations to persist despite control interventions.

The susceptibility of *Ae. aegypti* L3–L4 larvae to temephos was evaluated, revealing a positive dose–response relationship in the studied population, with a calculated LC50 of 0.034 μg/mL, which indicates that this concentration results in 50% mortality in larvae exposed for 24 h. In comparison, the LC50 for the Rockefeller strain was 0.0185 μg/mL, indicating that the field strain is approximately 1.84 times more resistant. This difference suggests that prolonged exposure to insecticides in the field population may have favored the selection of resistance mechanism.

This relatively high LC_50_ value suggests that a concentration higher than the discriminant larvicide of 0.012 μg/mL is required [65]. This finding may explain the challenges in the chemical control of larval populations and underscores the need to consider alternative control methods. For instance, in the department of Atlántico, Colombia, the continued use of temephos has led to high levels of resistance to the discriminant concentration (0.012 μg/mL) in *Ae. aegypti* in some municipalities, showing significant selection pressure and dominance of resistance alleles in the population [66]. Resistance to temephos has been reported to be associated with the overexpression of carboxylesterase, enzymes linked to resistance to organophosphates, carbamates, and, to a lesser extent, pyrethroids [67]. These enzymes can contribute to resistance through the hydrolysis of the insecticide, thus reducing its effectiveness [68,69].

As previously mentioned, the LC_50_ for temephos in our field population of *Ae. aegypti* is 0.034 μg/mL, while Grisales et al. [70] reported an LC_50_ of 0.066 μg/mL for a resistant population in Cúcuta, Colombia, representing a 15-fold increase compared to the LC_50_ from a laboratory susceptible strain. This difference suggests that our population is more susceptible to temephos compared to the population of Cúcuta. The findings of Grisales et al. [70] also indicate that resistance in Cúcuta could be associated with metabolic mechanisms, particularly with cytochrome P450 oxidases.

In México, Dávila-Barboza et al. [71] highlight widespread resistance to temephos in *Ae. aegypti*. Their study found that 30% of the evaluated populations showed high resistance, with the resistance level exceeding 10 times the discriminant concentration of 0.012 μg/mL. Similarly, resistance to this organophosphate has been documented in Brazil, where resistant populations have circulated across approximately half of the country since 2002–2003, leading to a reduction in larval mortality to below 80% since 2004 [72]. These findings emphasize the urgent need for continuous surveillance, the diversification of larvicide use (both chemical and bacterial), and the implementation of integrated management strategies to mitigate resistance and prevent its emergence in populations that remain susceptible.

On the other hand, when evaluating the effect of the different concentrations of temephos on *Ae. aegypti* larvae, other studies have observed that increasing the insecticide concentration leads to an extension of the larval development time and reduced adult longevity compared to the control strain [73,74].

The evaluation of deltamethrin susceptibility in adult females of *Ae. aegypti* showed a decrease in mortality over time. This suggests that some mosquitoes have the ability to recover from the knockdown effect induced by deltamethrin. In addition, the mortality rate of less than 98% in the field strain of *Ae. aegypti* aligns with previous reports of deltamethrin-resistant populations of *Ae. aegypti* worldwide [75]. Resistance to pyrethroids, including deltamethrin, has been widely linked to voltage-gated sodium channel mutations that cause knockdown resistance (*kdr*) and increased enzyme activity [76]. Moreover, certain bacterial symbionts, such as *Serratia oryzae* and *Acinetobacter junii*, have been shown to contribute to increased deltamethrin resistance [20,31].

When comparing our results with those of Pareja-Loaiza et al. (77) in northern Colombia, variability in susceptibility to deltamethrin (10 μg/flask) was observed through differences in mortality rates between locations. This was related to the presence of kdr alleles (V1016I, F1534C, V410L) and alterations in enzyme activity, with some areas showing high mortality and others resistance in *Ae. aegypti* populations.

The resistance of populations of *Ae. aegypti*, both in larvae and adults, in the face of insecticides highlights the need to adjust vector control strategies to effectively address resistance in different locations. This is particularly crucial in the department of Caquetá, where the increasing incidence of dengue in the region is being mitigated through various control measures. In Colombia, several integrated management strategies are being implemented to control *Aedes*, which not only include insecticide use but also focus on preventing and controlling breeding sites, as well as the promotion of good practices in water management and biological control [77]. However, it is essential to continue strengthening these strategies by promoting community education programs on dengue transmission and prevention and engaging the community in surveillance and control [78]. An integrated and adaptive approach is crucial to mitigate resistance and reduce dengue incidence in the region.

After analyzing insecticide resistance in *Ae. aegypti* and selecting resistant individuals, we obtained the culturable fraction of the intestinal microbiota from larvae and field-collected females. Although this fraction represents less than 5% of the total microbial community [79,80], the in vitro approach is valuable for bioprospecting, as it allows the identification of bacteria with biotechnological potential [23]. These bacteria could serve as potential targets in the search for molecules capable of modulating or participating in insecticide resistance mechanisms as well as in degradation processes.

However, the low representation of the culturable fraction limits this approach and can introduce bias, as conventional culture media may favor or inhibit the growth of certain bacteria [81]. This has prompted the need for alternative culture methods [82,83] and, currently, the implementation of culturomics [84], which combines culture conditions with methods such as MALDI-TOF (Matrix-Assisted Laser Desorption/Ionization Time-of-Flight) [85,86] and 16S rRNA gene sequencing, facilitating the identification of bacterial species in mosquitoes, including symbionts [87]. These methods could complement the results obtained in the current study in future research.

The results suggest that exposure to the evaluated insecticides could influence the composition of the gut microbiota of *Ae. aegypti*. In agreement with these findings, previous studies have shown that some bacterial communities in mosquito guts can affect their susceptibility to insecticides [88]. These interactions were first reported in agricultural pests, such as *Plutella xylostella*, which showed differential resistance to insecticides like chlorpyrifos and fipronil [89]. In these studies, the predominance of *Lactobacillales* in resistant lines indicated a potential link between the gut microbiota and insecticide resistance [89]. Similarly, it has been reported that suppressing the gut microbiota of *Ae. aegypti* generates a decrease in survival when exposed to permethrin, demonstrating that symbiotic bacteria can confer adaptive advantages to mosquitoes against insecticides [88].

Similarly, Soltani et al. [90] analyzed the effects of symbiotic bacteria on enzyme activity related to the resistance of *An. Stephanie* to temephos. Their findings revealed that the presence of these bacteria correlates directly with resistance to temephos. It was also observed that the enzymatic activity of the resistant insects resembled the susceptible phenotype when antibiotics were added. Although few species of insecticide-degrading bacteria have been reported in *Aedes* species, it has been confirmed that certain symbiotic bacteria such as *Serratia oryzae* promote the metabolic detoxification process through their enzymes and host systems [20]. These results underscore the potential of symbiotic bacteria as key players in resistance mechanisms, reinforcing the hypothesis that gut microbiota can significantly affect mosquito susceptibility to insecticides.

Our study focused on characterizing the microbiota of field *Ae. aegypti* under three conditions: untreated females (UFTs), deltamethrin-resistant females (DRFs), and temephos-resistant larvae (TRL). Molecular characterization of the gut bacteria in both larvae and adult females of *Ae. aegypti* aligns with previous studies using pyrosequencing of 16S rRNA, which reported a higher diversity of aerobic and facultative anaerobic Gram-negative bacteria in *Ae. aegypti* and other mosquito species [91,92]. Notably, no Gram-positive bacteria were identified in the insecticide-treated larvae and females, although they accounted for 1% of the gut microbiota in untreated females. One aspect to consider in this study is the exclusive use of LB medium. Even though it is a nutrient-rich medium, it may have restricted the identification of other bacterial species present in the *Ae. aegypti* gut microbiota. Previous research suggests that the use of culture media with different nutrients, along with diverse culture conditions, is a strategy that can be implemented to obtain a more comprehensive understanding of culturable bacterial diversity [31].

Taxonomic assignation using sequencing of the 16S rRNA gene and the *gyrB* gene markers revealed significant variations in the bacterial community of *Ae. aegypti* gut across different treatments. These findings suggest a possible association between insecticide resistance and changes in the gut microbiota of *Ae. aegypti*, consistent with previous research indicating that exposure to insecticides can induce shifts in the gut microbiota composition [29,30], favoring the growth of specific bacteria capable of degrading insecticides [31].

Three bacterial phyla were identified: Bacteroidetes, Firmicutes, and Proteobacteria, the latter being the most predominant (85.71%) in all treatments. This is consistent with previous studies that have reported Proteobacteria as the dominant phylum in the gut microbiota of *Ae. aegypti*, *Ae. albopictus*, and other mosquito species collected in the field, including *Anopheles gambiae* [30,92,93,94]. Firmicutes was the least representative phylum (3.6%), found only in UFTs, while Bacteroidetes (10.7%) was present only in DRFs and TRL. These findings coincide with those of Mancini et al. [33], who identified Proteobacteria, Bacteroidetes, Firmicutes, and Actinobacteria as the most abundant phyla in adult mosquitoes. Similarly, studies in fourth-instar larvae have identified Actinobacteria, Bacteroidetes, and Proteobacteria as the predominant phyla in the total microbiota community [20,91]. However, Actinobacteria was not detected in our study. This absence could be related to the limitations of the culture medium used, specific environmental conditions, or biological factors that influence the composition of the microbiota in the mosquito populations studied, including the presence of resistant phenotypes.

The dominance of Proteobacteria in the *Ae. aegypti* gut microbiota suggests a central role for this phylum, likely due to its ability to adapt to various environmental and metabolic conditions [6]. Proteobacteria are also the primary symbionts in many insect species, showing greater efficiency in colonizing and proliferating within new hosts than other bacterial groups [95]. Furthermore, metataxonomic analysis of the 16S rRNA gene has revealed that the dominance of this phylum extends beyond the gut, also being prevalent in the salivary glands and reproductive tracts of several mosquito species [33].

The genera *Enterobacter*, *Bacillus*, *Cedecea*, *Elizabethkingia*, *Serratia*, *Chryseobacterium*, and *Acinetobacter*, identified in our study, have also been reported in the gut of *Aedes* species and other mosquitoes [35,96]. *Enterobacter* is the predominant genus of culturable gut bacteria in both *Ae. albopictus* and *Ae. aegypti*, as demonstrated by molecular characterization of the microbiota in Arunachal Pradesh, India [96]. In the present study, *Enterobacter* was the most abundant genus in both UFTs and TRL, indicating its consistent presence regardless of insecticide treatment under laboratory conditions. However, it was absent in DRFs, suggesting a possible differential sensitivity to deltamethrin or microbial competition that may displace *Enterobacter* in the presence of this insecticide. Similarly, Dada et al. [29] found lower bacterial diversity in *An. albimanus* populations that are resistant to fenitrothion compared to susceptible populations, which could be attributed to the selection of bacteria that utilize the insecticide as a carbon and energy source. Their study also found that *Klebsiella* was enriched in the resistant strain, while the relative abundance of *Enterobacter* decreased.

*Enterobacter* is a genus known for its high genetic similarity between species and horizontal gene transfer, which complicates its precise classification [97,98]. In this study, sequencing with the gene marker *gyrB* facilitated taxonomic resolution, allowing the identity of several species to be confirmed, including *Enterobacter asburiae* and *Enterobacter mori*, present in both UFTs and TRL, and *Enterobacter cloacae*, which is specific to TRL.

Various studies have shown that bacteria such as *Bacillus cereus*, *Enterobacter asburiae*, and *Pantoea agglomerans* can degrade acephate, an organophosphate, to which *Plutella xylostella* has developed resistance [99]. *Enterobacter asburiae* has also been reported as a degrader of the organophosphate chlorpyrifos at concentrations of 250 mg/L, suggesting its possible use in the elimination of pesticides in contaminated soils [100]. Likewise, *Enterobacter cloacae* has demonstrated the ability to degrade DDT under aerobic conditions, highlighting its potential use in bioremediation of contaminated soils [101]. Additionally, a strain of *Enterobacter cloacae*, commonly found in the midgut of mosquitoes, can stimulate the expression of serpins, especially SRPN6, thereby enhancing their immune response of *Anopheles stephensi* against *Plasmodium falciparum* [102]. In contrast, *Enterobacter mori* has been associated with the exudate of *Parthenium hysterophorus* roots and influences the olfactory oviposition responses of *Anopheles gambiae*, suggesting important implications for mosquito ecology and control, especially in areas where *Anopheles gambiae* is a vector for disease [103].

*Acinetobacter* and *Serratia*, genera identified exclusively in the TRL treatment, have demonstrated the ability of some bacterial species to degrade organophosphate and pyrethroid insecticides, both in vitro and in contaminated soils, including compounds such as deltamethrin and cypermethrin [104,105]. *Serratia* sp., a gut symbiont, was the predominant bacterium in the midgut of *Ae. aegypti* larvae, suggesting that it may have a competitive advantage over other bacteria in this microenvironment [32]. Additionally, species like *Serratia marcescens* increase mosquito susceptibility to arboviruses by secreting the Sm Enhancin protein, which damages the physical barrier to dengue viruses, facilitating their infection and spread [106]. Similarly, Coon et al. [91] reported that *Chryseobacterium*, another genus present in the intestinal community of mosquitoes throughout all their life stages, as well as specific strains of *Chryseobacterium* sp. in various geographical locations, support their mutualist role [107]. Likewise, this bacterium contributes beneficially by facilitating the axenic larval development of mosquitoes [108]. A recent study also showed that the strain *Chryseobacterium* sp. BSC2-3 can degrade the pesticide carbofuran, transforming it into 3-hydroxy carbofuran, thus contributing to bioremediation processes that mitigate soil and water contamination [109].

The predominance of *Elizabethkingia* sp. in deltamethrin-resistant *Ae. aegypti* females highlights its symbiotic role, present in the midgut of laboratory-reared mosquitoes [110]. In a previous study, *Elizabethkingia* spp. (Flavobacteriaceae) were found exclusively in sugar-fed *Ae. aegypti* females and were absent after blood feeding [111]. Similarly, it has been demonstrated that *Elizabethkingia meningoseptica* becomes abundant in *Ae. aegypti* after exposure to deltamethrin [24].

*Cedecea*, the most abundant genus in the deltamethrin-resistant females (DRFs) treatment, has been reported to exhibit several co-exclusion relationships with dominant bacterial taxa such as *Asaia*, *Pseudomonas*, and *Enterobacter* in vectors including *Ae. aegypti*, *Ae. albopictus*, and *Culex quinquefasciatus,* observed in both field and laboratory mosquitoes [112]. In this study, co-exclusion was also observed, which may be attributed to competition for resources or ecological niches, where the presence of certain bacterial groups limits the availability of resources for others. Alternatively, it could be due to the production of inhibitory compounds by some bacteria that negatively affect the growth of others. Notably, *Cedecea neteri* is an isolated symbiont from *Aedes* that alters biofilm formation and reduces intestinal colonization in *Ae. aegypti* mosquitoes [113]. While *Cedecea neteri* is resistant to various antibiotics, including β-lactams and colistin [114], it has not been reported to degrade insecticides.

Conversely, *Bacillus aerius*, found exclusively in untreated females (UFTs), may be associated with the absence of selective pressure by insecticides under laboratory conditions for this group. However, it is possible that in the field, these mosquitoes have been exposed to insecticides. Furthermore, the biodegradation capacity of *Bacillus* species, such as the *B. subtilis* 1D strain, which degrades cypermethrin without producing toxic end products, highlights the potential of this genus for bioremediation of soils and waters contaminated with pesticides [115].

*Ae. aegypti* microbiota varies by sex, developmental stage, diet, and environmental factors [116]. In general, bacterial diversity at the species level is lower in adults than in larvae. This is consistent with other studies that show lower bacterial diversity in the adult stage of *Aedes* and other insects, which is attributed to the intestinal renewal that occurs during metamorphosis from pupae to adults [91,117]. These bacteria are acquired from the aquatic environment during larval development, although vertical transmission has also been documented [94]. Several studies have shown that larval-stage microbiota composition is similar to that of their aquatic environment [118], and some bacteria can persist into their adult state [119]. In adults, feeding behavior also modulates the gut microbiota, increasing its bacterial load but decreasing its diversity [120].

It is important to consider that rearing conditions and mosquito origin impact the composition of the gut microbiota [121]. Untreated *Ae. aegypti* females, reared with water from the natural site, had more naturally occurring bacteria than insecticide-treated females reared entirely in the laboratory. Controlled laboratory environments can significantly alter the composition and diversity of the microbiota compared to natural conditions, where larvae are exposed to a wide variety of physical, chemical, and biological factors [93,121]. Baltar et al. [122] demonstrated that bacterial richness and diversity in *Ae. albopictus* populations in Rio de Janeiro, Brazil, were higher in field mosquitoes than in those reared for a single generation in the laboratory (F1), highlighting how these factors can influence the composition of the intestinal microbiota.

An important aspect of the microbiota in insecticide resistance is its role in mosquito survival after insecticide exposure. Suppression of the microbiota with antibiotics, such as penicillin and streptomycin in axenic mosquitoes, has been shown to reduce mosquito survival when exposed to permethrin [88]. This decrease in survival is particularly associated with the elimination of bacteria from the *Enterobacteriaceae* family, suggesting their potential role in conferring insecticide resistance [88]. Similarly, Arévalo et al. found that axenic adult mosquitoes exhibit increased susceptibility to lambda-cyhalothrin, indicating that specific bacteria or their metabolites may contribute to the insecticide-resistant phenotype observed in Colombian populations of *Ae. aegypti*.

Our findings highlight the importance of understanding gut microbiota diversity in *Ae. aegypti*, particularly regarding the selection of field populations resistant to insecticides like temephos and deltamethrin. On this matter, variability in bacterial composition could not only influence mosquito biology but also the effectiveness of control strategies. This study provides a solid basis for future research on the interactions between microbiota and insecticide resistance. It also paves the way for the exploration of metabolic bacterial traits which could facilitate the development of relevant biological control strategies.

Within the context of vector control, the role of the intestinal microbiota in modulating insecticide resistance levels must be considered along with its key role in bioprospecting processes aimed at bioremediating the environmental residues and bioaccumulation caused by the widespread use of chemical insecticides. In the framework of integrated vector management, local authorities are encouraged to consider alternatives such as insecticide rotation, based on resistance patterns associated with microbiota [123]. This approach could help minimize the selection of resistant mosquito populations. Additionally, microbiome studies highlight the potential use of endosymbionts such as *Wolbachia* [124], which represent cost-effective, sustainable, and long-term biological control strategies for managing arbovirus transmission in regions such as Caquetá.

## 5. Conclusions

This study revealed a high positivity in ovitraps located in the different neighborhoods in Florencia, Caquetá, Colombia, which indicates favorable conditions for the proliferation of *Ae. aegypti*. The notable presence of mosquitoes in indoor spaces highlights the high risk of ABV transmission.

Under the conditions evaluated, it was determined that the population of *Ae. aegypti* in Florencia, Caquetá, presents resistance to temephos and suggests a possible resistance to deltamethrin. This resistance could be related to the increase in the number of cases in the region. In addition, these findings highlight the need to adjust control strategies, implement insecticide rotation in integrated management programs, and conduct periodic monitoring to effectively regulate resistance.

Among the seven genera identified, the abundance of *Serratia* in resistant larvae and the predominance of *Cedecea* in resistant females suggest a possible association with observed symbiont-mediated resistance. These bacteria may influence the ability of *Ae. aegypti* larvae and adults to resist insecticides. However, the possibility that the other genera identified may also have a similar potential in the degradation of insecticides or the modulation of resistance has not been ruled out. Therefore, it is crucial to conduct further studies to explore the role of all bacterial genera present and their potential contribution to *Ae. aegypti* insecticide resistance.

## Figures and Tables

**Table 1:** Taxonomic classification of bacteria isolated from the gut of *Ae. aegypti* into UFTs, TRL, and DRFs, according to their similarity to *gyrB* gene sequences recorded in the GenBank database.

Code Sequencing	Isolate Code	Origin *	NCBI–GenBank Identifier	NCBI Code	% Similarity
J1G	P1D1C1	UFTs	*Enterobacter asburiae*	KP345905.1	99.02%
J3G	P1D3C1	UFTs	*Bacillus aerius*	CP133585.1	99.52%
J4G	P2D2C1	UFTs	*Enterobacter asburiae*	CP056716.1	94.46%
J10G	P31D2	TRL 0.01 µg/mL	*Chryseobacterium gleum*	LR134289.1	97.11%
J11G	P23D2	TRL 0.03 µg/mL	*Enterobacter cloacae*	OL364945.1	98.57%
J16G	P15D2	TRL 0.05 µg/mL	*Acinetobacter* sp.	CP028561.1	96.36%
J17G	P32D3	TRL 0.02 µg/mL	*Chryseobacterium gleum*	LR134289.1	96.75%
J19G	P43D4	TRL 0.03 µg/mL	*Enterobacter asburiae*	CP129027.1	99.14%
J28G	P43D3	TRL 0.03 µg/mL	*Enterobacter asburiae*	CP129027.1	99.11%
J29G	P2HD2	DRFs	*Cedecea neteri*	CP134767.1	97.03%
J31G	P1HD2	DRFs	*Cedecea neteri*	MN329097.1	99.49%

* Untreated females (UFTs), deltamethrin-resistant females (DRFs), and temephos-resistant larvae (TRL).

## Data Availability

We have provided all data in the Supplementary Materials.

## Supplementary Materials

The following supporting information can be downloaded at: https://www.mdpi.com/article/10.3390/insects16020181/s1. Figure S1: Susceptibility bioassays of insecticides and intestinal tissue of *Ae. aegypti.* Figure S2: Diagram of bacterial culture and CFU estimation from *Ae. aegypti* larvae and females. Figure S3: Ovitrap positivity rate (%) in the four sampled neighborhoods of Florencia, Caquetá, Colombia. Figure S4: Total number of viable and non-viable eggs of *Aedes* spp. Figure S5: Colony-forming units per milliliter (CFU/mL) and counts of bacterial cultures obtained from *Ae. aegypti* guts from the different treatments. Figure S6: Gram-staining-based characterization of bacterial isolates. Figure S7: PCR amplification of the internal transcribed spacer (ITS), 16S rRNA gene, and *gyrB* gene. Table S1: Information on bacterial isolates of *Ae. aegypti*, log colony-forming units, and locality where biological material was collected. Table S2: Bacterial isolates of *Ae. aegypti* from different treatments and sequences reported in GenBank.
