# Supplementary material for: Diversity of Gut Bacteria of Field-Collected Aedes aegypti Larvae and Females, Resistant to Temephos and Deltamethrin
Source: Insects. 2025 Feb 8;16(2):181. doi: 10.3390/insects16020181 (PMC11856030; doi:10.3390/insects16020181)
Supplement: Supplementary file 1 [file insects-16-00181-s001.zip › insects-3419466-supplementary.pdf]

Diversity of gut bacteria of field-collected *Aedes aegypti* larvae and females, resistant to temephos and deltamethrin

Supplementary Materia

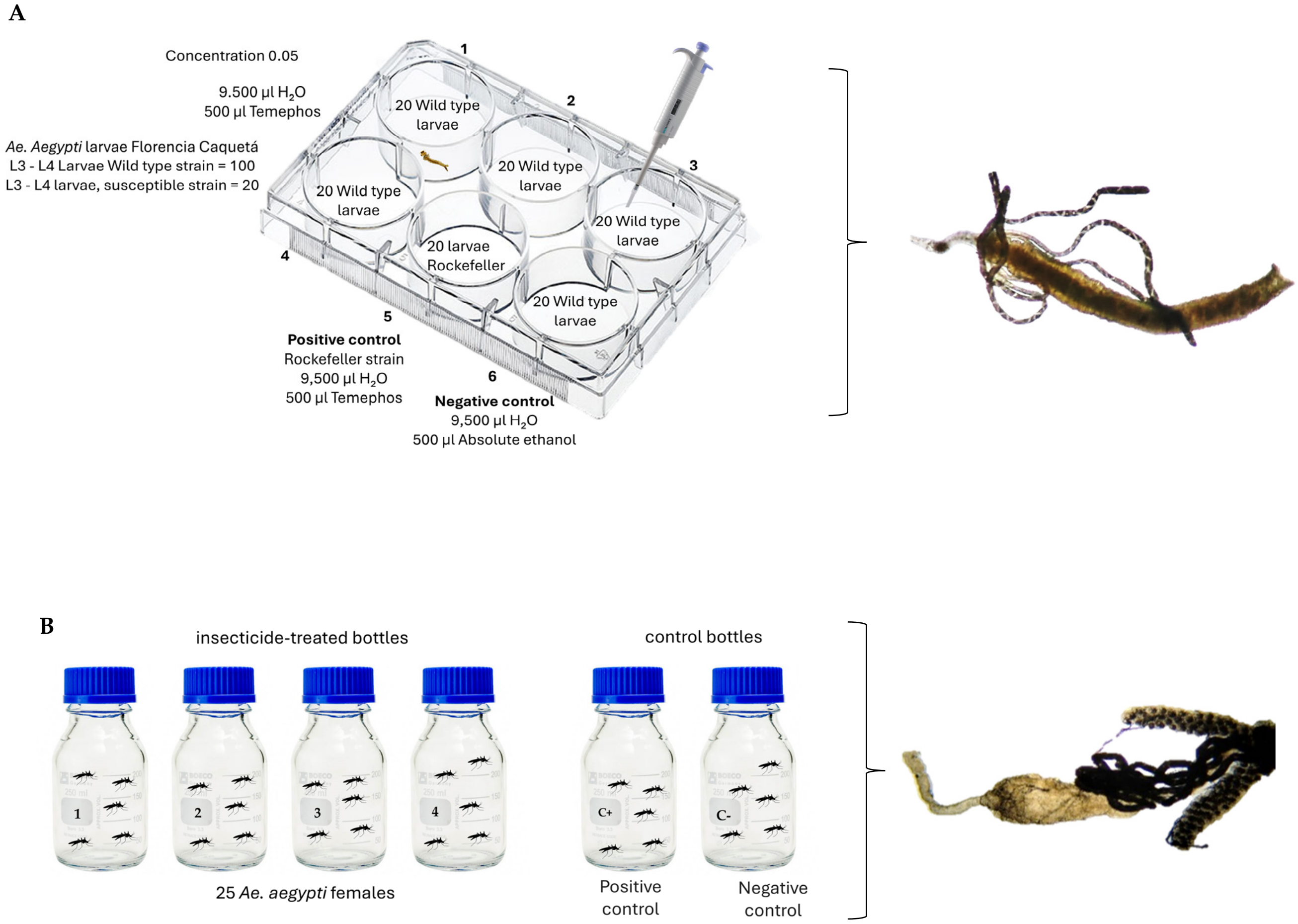

Supplementary Figure S1. Susceptibility bioassays of insecticides and intestinal tissue of *Ae. aegypti* **(A)** Bioassay design for resistance to temephos with L3–L4 larvae of *Ae. aegypti* in six-well plate. 4 replicates are included, with positive control (Rockefeller strain) and negative control (field strain). On the right is shown the intestinal tissue of L3–L4 larvae of *Aedes aegypti*. **(B)** Design of the deltamethrin resistance bioassay in *Ae. aegypti* females in Wheaton bottles. Four replicates were included, along with a positive control (Rockefeller strain) and a negative control (field strain). On

the right, intestinal tissue from females resistant to deltamethrin is observed.

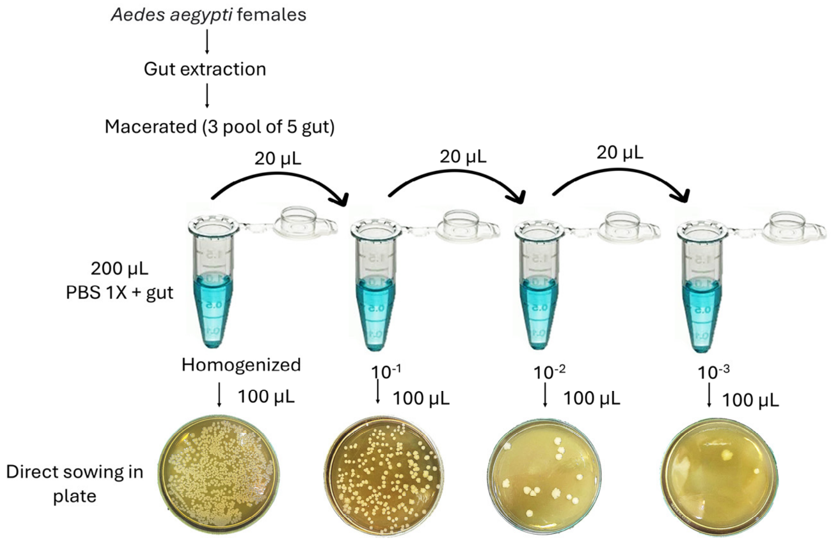

Supplementary Figure S2. Representative diagram illustrating the process of obtaining bacterial cultures and estimating bacterial load (CFU) from the intestinal homogenate of *Ae. aegypti* larvae and females.

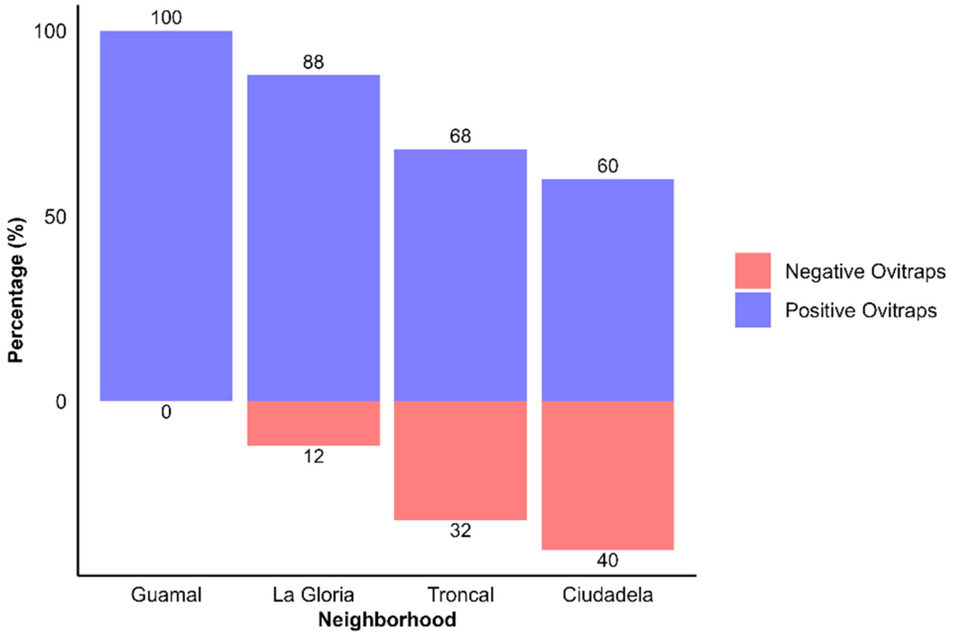

Supplementary Figure S3. Ovitrap positivity rate (%) in the four sampled neighborhoods of Florencia, Caquetá, Colombia. The percentages of positive and negative ovitraps in each location are presented.

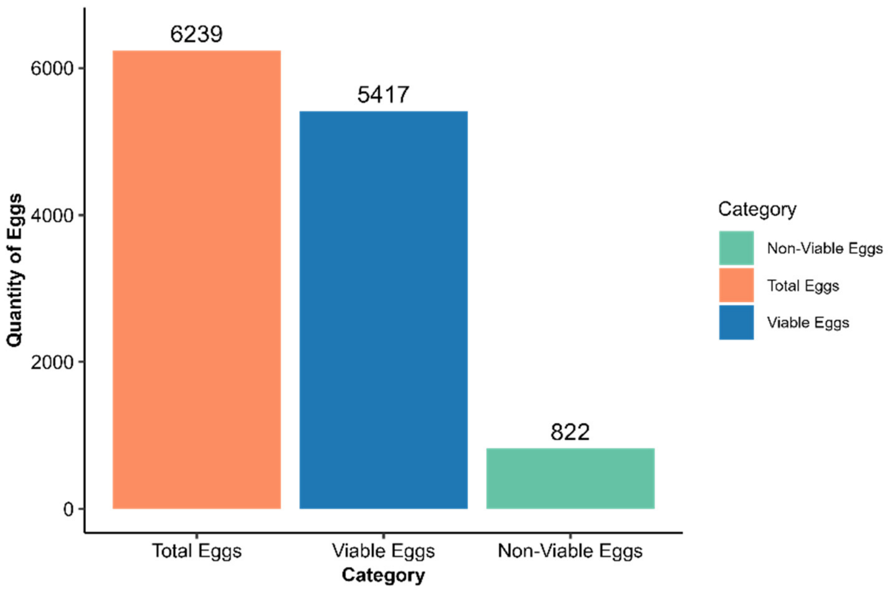

Supplementary Figure S4. Total number of viable and non-viable eggs of *Aedes* spp. collected in the four neighborhoods of Florencia, Caquetá, Colombia.

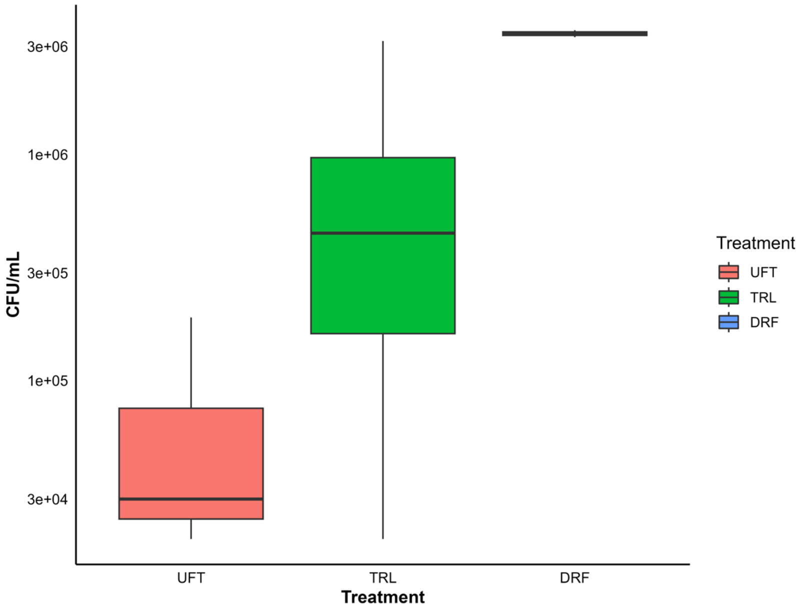

Supplementary Figure S5. The count of colony-forming units per milliliter (CFU/mL) of bacterial cultures obtained from the intestines of *Ae. aegypti* from different treatments: untreated females (UFT) (8×10^4), temephos-resistant larvae (TRL) (9.4×10^5), and deltamethrin-resistant females (DRF) (3.42×10^6). Statistical analysis showed significant differences in CFU/mL count between treatments (P value= 0.0195). It was found that the DRF treatment had a significantly higher CFU/mL compared to UFT (P value = 0.0162). However, no significant differences were observed between TRL and UFT (P value = 0.1017) or between DRF and TRL (P value = 0.1606)

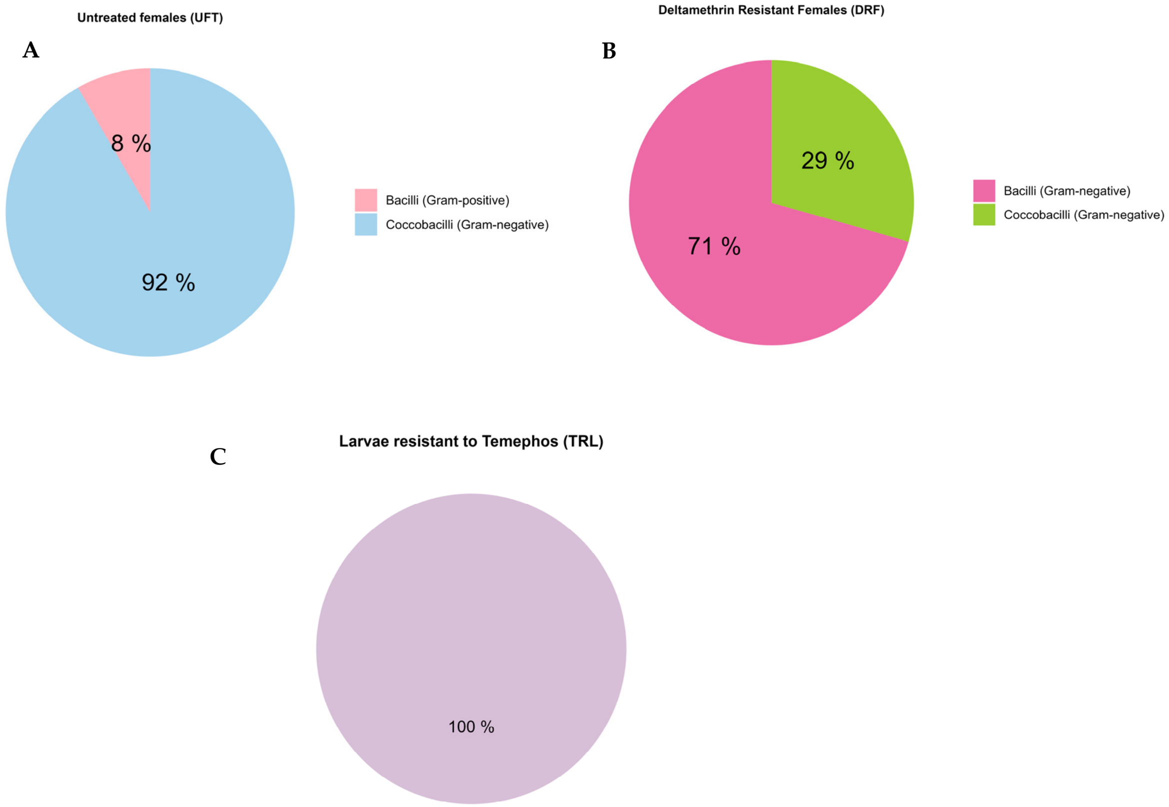

Supplementary Figure S6. Characterization of bacterial isolates according to Gram staining. **(A)** Percentage of gram-negative and gram-positive bacteria in isolates from untreated females. **(B)** Isolates from deltamethrin-resistant females. **(C)** Isolates from L3-L4 larvae of Temephos-resistant *Ae. aegypti.*

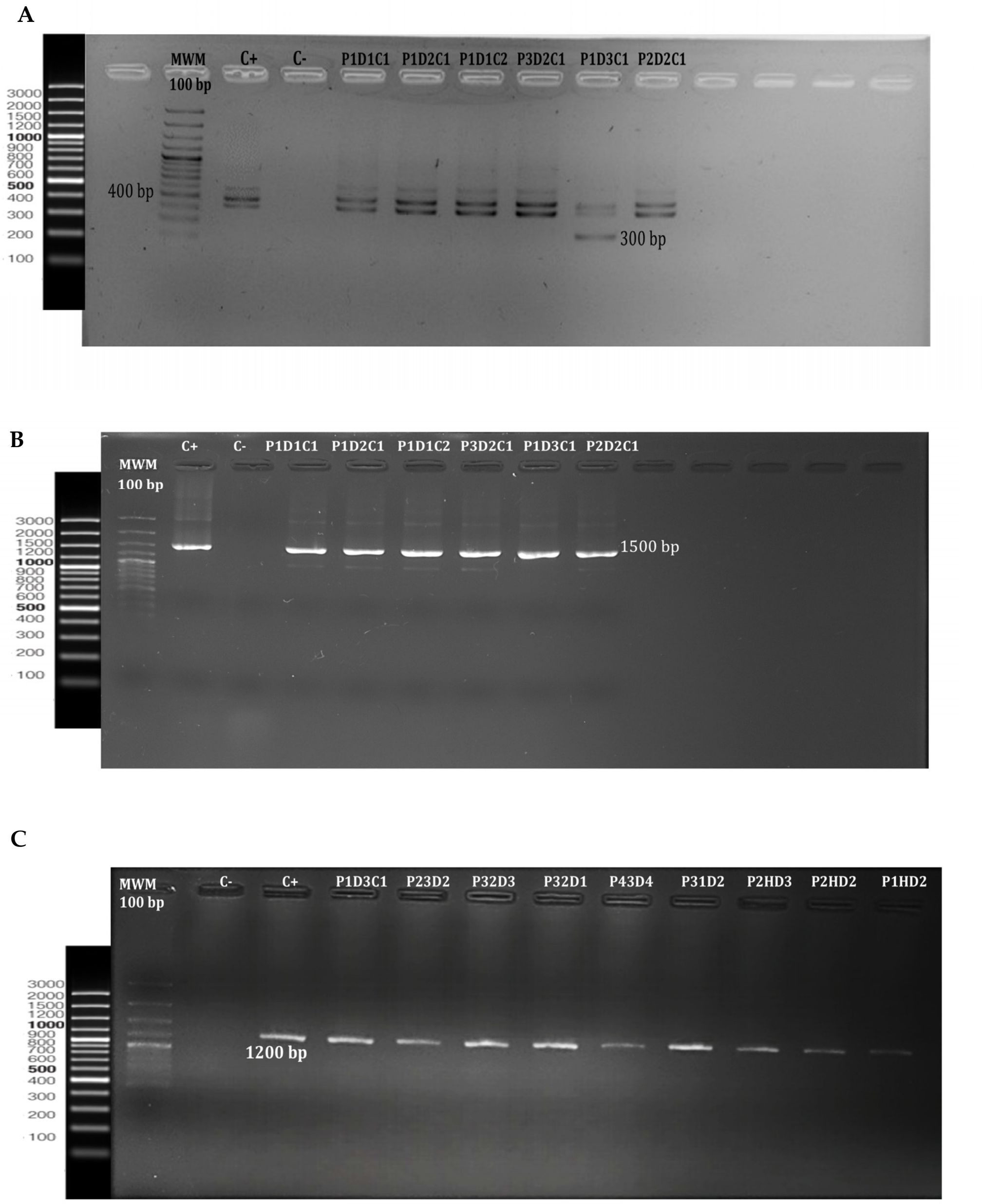

Supplementary Figure S7. PCR amplification. **(A)** Amplification of the transcribed internal spacer (ITS), visualized by 2% agarose gel electrophoresis. Controls: C+ (*Enterobacter* sp). C- (Ultrapure water). Samples: P1DC1, P1D2C1, P1D1C2, P3D2C1, P1D3C1, P2D2C1, bacterial isolates from intestines of female *Ae. aegypti* from Florencia, Caquetá, not treated with insecticide **(B)** Amplification of the 16S rRNA gene, visualized in 1.2% agarose gel. Controls: C+ (*Bacillus* sp*)*. C- (Ultrapure water). Samples: P1DC1, P1D2C1, P1D1C2, P3D2C1, P1D3C1 and P2D2C1, bacterial isolates from intestines of female *Ae. aegypti*, not treated with insecticide. **(C)** Amplification of the *gyrB* gene, visualized in 1% agarose gel. Controls: C+ *(Bacillus* sp). C- (Ultrapure water). Samples: P1DC3, bacterial isolate from intestines of *Ae. aegypti* females, not treated with insecticide, P23D2, P32D3, P32D1, P43D4, P31D2 from Temephos-resistant *Ae. aegypti* larvae and P2HD3, P2HD2 and P1HD2, Deltamethrin-resistant females.

Table S1: Information on bacterial isolates of *Ae. aegypti*, colony forming units, and locality where biological material was collected.

Isolated code	Species	Stadium	Treatment	Number guts	CFU/mL	Isolated number	Location
P1D1C1	*Ae. aegypti*	Female	Untreated	5	3.00E+04	1	Florencia Caquetá, Colombia
P2D3C1					2.00E+04	3	
P1D3C1					1.90E+05	3	
P11D2		Larvae	Resistant larvae		2.00E+05	2	
P31D2					2.00E+04	2	
P24D2					1.08E+06	2	
P32D3					4.20E+05	3	
P43D4					3.05E+06	4	
P31D2					8.70E+05	2	
P22D2					4.50E+05	2	
P32D2					1.30E+05	2	
P12D3					5.40E+05	3	
P34D3					7.00E+04	3	
P43D3					3.17E+06	3	
P2HD2		Female	Resistant female		3.30E+06	2	
P2HD3					3.54E+06	3	

Table S2: Bacterial isolates of *Ae. aegypti* from different treatments and sequences reported in GenBank.

Number	Treatment	Isolated code	Bacterial species	NCBI-GenBank accession number: 16S rRNA gene	NCBI-GenBank accession number: *GyrB* gene
1	UFT	P1D1C1	*Enterobacter asburiae*	PP708950	
2		P1D2C1	*Enterobacter mori*	PP731791	
3		P1D3C1	*Bacillus aerius*		PQ538553
4		P2D2C1	*Enterobacter asburiae*		PQ538554
5		P2D3C1	*Enterobacter mori*	PP732996	
6		P1D3C2	*Enterobacter asburiae*	PQ083278	
7		P2D2C2	*Enterobacter* sp.		
8		P2D1C2	*Enterobacter mori*	PQ083526	
9	TRL	P11D2	*Enterobacter* sp.		
10		P31D2	*Chryseobacterium gleum*		PQ538555
11		P23D2	*Enterobacter cloacae*		PQ538556
12		P31D2	*Serratia* sp.		
13		P32D1	*Enterobacter* sp.		
14		P22D3	*Serratia* sp.		
15		P24D2	*Enterobacter mori*	PQ083832	
16		P15D2	*Acinetobacter* sp.		
17		P32D3	*Chryseobacterium gleum*		PQ538557
18		P24D2	*Enterobacter* sp.		
19		P43D4	*Enterobacter asburiae*		PQ538558
20		P31D2	*Serratia* sp.	PQ083846	
21		P22D2	*Serratia* sp.		
22		P32D2	*Serratia* sp.		
23		P12D3	*Serratia* sp.		
24		P34D3	*Enterobacter* sp.		
25		P43D3	*Enterobacter asburiae*		PQ538559
26	DRF	P2HD2	*Cedecea neteri*		PQ538560
27		P2HD3	*Elizabethkingia* sp.		
28		P1HD2	*Cedecea neteri*		PQ538561
